# Prediction of the Influential Factors on Eating Behaviors: A Hybrid Model of Structural Equation Modelling-Artificial Neural Networks

**DOI:** 10.1155/2020/4194293

**Published:** 2020-05-18

**Authors:** Maryam M. Kheirollahpour, Mahmoud M. Danaee, Amir Faisal A. F. Merican, Asma Ahmad A. A. Shariff

**Affiliations:** ^1^Institute of Advanced Studies (IAS), University of Malaya, Kuala Lumpur 50603, Malaysia; ^2^Department of Social and Preventive Medicine, Faculty of Medicine, University of Malaya, Kuala Lumpur 50603, Malaysia; ^3^Institute of Biological Sciences, Faculty of Science, University of Malaya, Kuala Lumpur, Malaysia; ^4^Center of Research for Computational Sciences and Informatics in Biology, Bioindustry, Environment, Agriculture, and Healthcare (CRYSTAL), University of Malaya, Kuala Lumpur 50603, Malaysia; ^5^Mathematics Division, Centre for Foundation Studies in Science, University of Malaya, Kuala Lumpur 50603, Malaysia

## Abstract

The importance of eating behavior risk factors in the primary prevention of obesity has been established. Researchers mostly use the linear model to determine associations among these risk factors. However, in reality, the presence of nonlinearity among these factors causes a bias in the prediction models. The aim of this study was to explore the potential of a hybrid model to predict the eating behaviors. The hybrid model of structural equation modelling (SEM) and artificial neural networks (ANN) was applied to evaluate the prediction model. The SEM analysis was used to check the relationship of the emotional eating scale (EES), body shape concern (BSC), and body appreciation scale (BAS) and their effect on different categories of eating behavior patterns (EBP). In the second step, the input and output required for ANN analysis were obtained from SEM analysis and were applied in the neural network model. 340 university students participated in this study. The hybrid model (SEM-ANN) was conducted using multilayer perceptron (MLP) with feed-forward network topology. Moreover, Levenberg–Marquardt, which is a supervised learning model, was applied as a learning method for MLP training. The tangent/sigmoid function was used for the input layer, while the linear function was applied for the output layer. The coefficient of determination (*R*^2^) and mean square error (MSE) were calculated. Using the hybrid model, the optimal network happened at MLP 3-17-8. It was proved that the hybrid model was superior to SEM methods because the *R*^2^ of the model was increased by 27%, while the MSE was decreased by 9.6%. Moreover, it was found that BSC, BAS, and EES significantly affected healthy and unhealthy eating behavior patterns. Thus, a hybrid approach could be suggested as a significant methodological contribution from a machine learning standpoint, and it can be implemented as software to predict models with the highest accuracy.

## 1. Introduction

**Figure 1:**
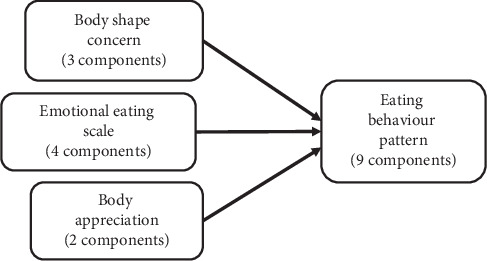
Conceptual frame of factors affecting EBP.

Recent studies indicated that behavioral factors, such as eating behavior pattern, emotional eating behavior, body shape concern, and body appreciation are the most influential factors which cause weight gain and obesity [1–3]. The present study aims to test a novel research model that processes three potential predictors of eating behavioral patterns, as a complement to the traditional research method from the perspective of analytical methodology improvement. This study among the few existing studies has applied machine learning approach to investigate the prediction model of eating behavior pattern considering the linear and nonlinear effects. According to the theory of planned behavior [4] and the pertinent literature, the model of eating behavior pattern and factors which have effect on that is made by applying four main constructs including body appreciation (BA), body shape concern (BSC), and emotional eating scale (EES) and eating behavior pattern (EBP). Therefore, in the present study, the conceptual model was conducted based on the theories and the related literature. The conceptual model consists of three exogenous (independent), including emotional eating scale, body shape concern, and body appreciation, and one endogenous (dependent variable) eating behavior patterns (see Figure 1). These factors are directly related to the assessment of eating behavior patterns (EBP) which also contribute to obesity [5]. Researchers found a strong relation between emotional eating and overeating and weight gain [6–8]. Body shape concern and body appreciation are among the factors directly related to the assessment of the impact of the body image on energy intake leading to obesity [9, 10].

The body shape concern is a construct assessing the individual perception of body shape, which entails behaviors, emotions, and beliefs associated with the self-perception [11]. This perception could be assessed using a psychometric scale such as BSCQ, which was recently adjusted to the number of three constructs and total of 34 items [12]. In addition, the impact of body appreciation on weight problems was recently studied [13]. The Body Appreciation Scale Questionnaire (BASQ) measures the positive perception of an individual on her/his own body, which was initially validated by Avalos et al. [14]. The BASQ covers positive opinions of the body, such as acceptance of the body despite its imperfections and respect and protection for the body [15]. This questionnaire consists of two components with 13 items. The emotional eating is known as “eating in response to a range of negative emotion to cope with negative effect” [16]. Also, the Emotional Eating Scale Questionnaire (EESQ) was revised to measure eating habits in response to specific emotions, including anger, anxiety, and happiness (four components and 18 items) [17]. Recently, the Eating Behavior Pattern Questionnaire (EBPQ) was validated which includes nine components with 51 items to use it against the traditional methods of dietary assessment for predicting fat and fiber intake in African American women with diverse socioeconomic status [5]. The EBPQ was used in epidemiologic studies to measure the relationship between the eating behavior patterns and health outcomes. EBPQ consisted of nine categories of healthy and unhealthy eating behaviors including low-fat eating, eating outside, snacking, planning ahead, cultural habits, healthy eating, sweets, emotional eating, and meal skipping.

Since obesity is known as one of the main causes of diabetes, cancer, and cardiovascular disease, modifying these main determinants of chronic diseases could decrease diet-related diseases [18].

To evaluate the suggested model, structural equation modelling (SEM) was applied. SEM is known as a pure technique to examine the causal and linear relationships in modeling the public health and behavioral science problems. Specially, this method is used to validate the questionnaire-based studies with 5-likert scale points [19]. In the current study, the partial least square-SEM (PLS-SEM) estimation method was applied because this method is able to evaluate the linear relationships in the prediction of complex models. Also, the importance of using the PLS-SEM methods is to validate the measurement model with several constructs (latent variables) and their indicators (items) as the outer model and to evaluate the causal relationship between the latent constructs (components) as the inner model through the path model analysis [20]. Therefore, in the first stage of this work, the measurement model of all constructs and their indicators was checked and then the causal relationship between three constructs BSC, BA, and EES and nine components of EBPQ were determined. Specially, the inner model in SEM analysis was involved in this analysis. However, SEM could only examine the linear relationships. Most of these factors could have a nonlinear effect on EBP. That is why more accurate methods were needed to evaluate this model in presence of nonlinear relationship among the variables (inner model).

In order to have accurate estimation, along with statistical approaches, the nonlinear methods such as neural networks are suggested. A large number of scholars have applied the combination of statistical methods and neural networks rather than using traditional statistical methods or neural networks separately [21–24]. A majority of these researchers have found that the hybrid model could achieve more accurate results with higher *R*^2^ (coefficient of determination) and lower level of error indexes, such as RMSE and MSE simultaneously [25–29].

To fill the gap of this study which is the presence of nonlinear relationships among the variables of the model, a modern generation of modeling such as a hybrid model is suggested to address the linear and nonlinear relationships in this complex model. In this case, the hybrid model of SEM and ANN analysis is considered. ANN is a machine learning approach which is known as a strong method to deal with the nonlinear relationships in complex systems such as the eating behavior prediction model [30]. However, ANN analysis is not suitable for evaluation of linear relationships due to its nonlinear inherent [31]. Therefore, a combination of both methods are complementary (e.g., SEM model and ANN model) [19]. In fact, our ability to accurately map the eating behavior models would allow us to develop and test predictions of eating-related disease occurrence. A neural network model is a complex structure of input and output signals, and neurons whose signals come from inputs move through hidden units and finally reach the output units. All feed-forward multilayer ANN have a feed-forward structure. By far, in addition to feed-forward networks, other types of ANN (e.g., self-organizing maps (SOM) and radial base function (RBF)) are extremely useful in resolving real problems, and thus are commonly used. Neurons in a feed-forward network typically have a separate layered topology. Usually, the function of the input layer is to introduce values of the input variables. Each of the output and hidden layer neurons is associated with all the units of the previous layer. Multilayer perceptron (MLP) involves a system of layered interconnected nodes, and it consists of one or more hidden layers. Thus, the MLP is also called a feed-forward neural network input layer that transmits an input vector to the network. MLP with feed-forward network topology is one of the most popular ANN architecture. However, deep learning and convolutional ANN are known as the most popular and modern ANN topology. The equation of perceptron is written as follows:

(1) $$\begin{array}{c} {\text{Net}}_{k}=\;\varphi \;\left(\sum_{i=1}^{n}w_{i}x_{i}+b_{k}\right), \end{array}$$

where Net_*k*_ is the output signal, *φ* represents the activation function, the number of connections to the perceptron is *n*, *x* = (*x*_1_,…, *x*_*n*_) is the value of the *i*th connection, *w*=(*w*_1_,…, *w*_*n*_) is the weight associated with the *i*th connection, and *b* shows the threshold.

Based on the objectives of the study, the following research question is formed: To what extent the hybrid model (SEM-ANN) could evaluate the model of body shape concern, body appreciation, and emotional eating behavior in prediction of the eating behavior patterns? The results of this study could be especially beneficial for decision makers of the future prevention program of eating disorders.

## 2. (SEM-ANN) Hybrid Model Literature

According to the related literature, many researchers in a variety of fields of marketing [32], forecasting [33], quality of life management [34], energy and environmental engineering [35], and hydrology [36] applied the hybrid approach. Most of these researchers claimed that the hybrid method could be superior to traditional statistical techniques because it can measure nonlinear relations by using different activity functions and layers of hidden nodes. However, there are a few studies in which a hybrid approach of ANN and SEM has been applied. Furthermore, there is still no study using hybrid models in the field of public health and eating behavior.

Recently, the SEM was used as a new method for predicting compound and linear models. Since SEM is only capable of evaluating the linear relationships, it may sometimes oversimplify the complexities involved in the complex models. Using nonlinear neural networks analysis can complement the weaknesses of the linear SEM analysis. Only few studies related to business and marketing have used the (SEM-ANN) hybrid model to improve the prediction performance. In one study by Sharma et al., the SEM-ANN model was used [37] to predict main factors that have an impact on the intention of students regarding academic use of Facebook. Both nonlinear and linear modeling were employed to examine Facebook adoption. At first, the SEM was used to test the proposed hypotheses, while in the next stage, exogenous variables were adopted as the model input. This neural network modeling was shown to help better recognize factors predicting application of Facebook in higher education. Similar studies used the same approach to check the customer loyalty and customer satisfaction in the field of economy [30]. The results showed that the SEM-ANN as a two-stage predictive analytic offers a more complete understanding and therefore may have a substantial methodological contribution. Moreover, ANN and SEM were combined to study the factors with impact on consumers' intention to adopt m-commerce [38]. Prediction performance of the hybrid model was revealed to be good by other studies [35, 39, 40].  Table 1 presents the latest research on the SEM-ANN hybrid model.

## 3. Materials and Methods

### 3.1. Ethics Statement

The ethical approval was obtained from the University of Malaya, Faculty of Medicine (UM.TNC2/RC/H&E/UMREC-63). Students who agreed to participate in the study were given a questionnaire package, including the information sheet explaining about the research and the consent form. The subjects were asked to complete the questionnaires individually.

### 3.2. Participants

The population was randomly selected from the University of Malaya (semester I and II, the years 2016 and 2017) through the multistage cluster random sampling technique [43] with diverse socioeconomic status and without known physical or mental illnesses. In this study, the University of Malaya with 17 faculties was chosen. First, five faculties were randomly selected based on the highest percentage of students enrolled in each faculty. Second, the portion size and the number of samples from different faculties were determined. Third, five departments were chosen randomly of each faculty and the number of classes of one semester were obtained from the administration office of each department. Fourth, the classes were chosen randomly, and finally the participants were chosen randomly from the local students. Considering power analysis [44], the sample size needed for structural equation modeling was calculated. Basically, the amount of *β*, *α*, number of latent variable, and the number of indicators were fixed in this study. Accordingly, by considering *β*=0.8, number of latent variable = 17, number of indicators = 106 items, and *α*=0.05, the least number of sample calculated for PLS-SEM is equal to 340.

### 3.3. Questionnaire

The questionnaire that was used in the current study included five parts. All items of the questionnaire were adopted, and minor corrections during psychometric analysis were applied.

  Part 1: a self-report demographic questionnaire (age, marital status (single or married), educational level (Bachelor, Master, or PhD degree, and income level for those working)
  Part 2: the Body Shape Concern Questionnaire (BSCQ) (34 items) [45]
  Part 3: the Body Appreciation Questionnaire (BAQ) (13 items) [14]
  Part 4: the Emotional Eating Scale Questionnaire (EESQ) (51 items), developed in [46]
  Part 5: the Eating Behavior Pattern Questionnaire (EBPQ) (18 items) [5]

Statistical analysis was performed using MATLAB R2018b and Smart PLS, ver.3.

### 3.4. Structural Equation Modelling

Structural equation modeling consisted of two important stages, the measurement model and the structural model. This is a cross-sectional study that is designed to examine the eating behavior risk factors among the university students. Regarding the setting of the SEM model, three constructs are defined as predictors (exogenous variables) in the model, including emotional eating scale (EES), body appreciation (BA), and body shape concern (BSC). Also, the nine categories of eating behavior patterns (EBP) are considered as dependent factors (endogenous variables). In SEM analysis, the measurement model was used to verify the convergent and discriminant validity. Since the power of SEM analysis is based on evaluation of the measurement model, at the first step, the SEM analysis was used to calculate the factor score through the measurement model. SEM analysis was then used after examining each latent variable with related indicators to find the contribution of each indicator to the related unobserved (latent) variable. Each latent variable was checked according to formative or reflective inherent in the first stage. All the constructs were reflective in the first order.

The causal relationship between BA, BSC, EES, and nine categories of EBP was examined through the structural model. The number 24 path coefficient which indicated the association of main constructs was examined. The bootstrapping was used to determine the significance of path coefficients, and the related variance explained by the model (*R*^2^) is attained. In this study, the partial least square- (PLS-) SEM was used as a preprocessor, in which the data were primarily preprocessed by the PLS-SEM (Smart PLS ver3), and then they were incorporated in the neural network model (using Matlab programing) rather than being used directly. At the final step, it was necessary to combine SEM and ANN. The architecture of ANN analysis needed input, output, and hidden layers. To conduct the hybrid model, the factor score for the main constructs obtained by SEM was incorporated as the input and output layers in ANN. In this case, the factor scores for the body appreciation, body shape concern, and emotional eating scale were used as an input variable in the neural network model. Hence, the factor score of nine categories of eating behavior patterns was used as an output to the neural network model. The number of hidden neurons was selected according to the criteria of ANN analysis. The best network was chosen according to the criteria.

### 3.5. Neural Network Approach

The current study aimed to investigate the appropriate hybrid method for measuring the association and the relation among the variables of interests. Accordingly, the factor score of three independent variables including BA, BSC, and EES was considered as input variables. Also, the factor scores of categories of EBP were applied as the output (target) in the architecture of the ANN model. The architecture of ANN includes defining the number of layers in the input, output, and hidden layers. In most of the cases, there is no way to determine the best number of hidden layer and the neurons in each of them, without training several networks and estimating the validation error of each [47]. Therefore, different number of selections of neurons in the hidden layer were examined. The selected number of neurons in the hidden layer was chosen between 10 and 20 because out of this interval, similar results were obtained that is why the numbers were not reported in this study. In ANN analysis, 70% of the data was used for neural network training and the rest (15%) was used to evaluate the accuracy of trained network's prediction, while 15% was used to validate the network performance. The optimal network was chosen according to the difference between test and training data sets. Also, multilayer perceptron (MLP) with feed-forward network topology was used. Moreover, Levenberg–Marquardt was applied as a supervised learning method for MLP training which was completely efficient among the gradient-based algorithm in fittings [48]. The tangent/sigmoid was used for the input layer according to the best performance for analyzing the complex model, and the linear model was applied for the output layer due to the fact that causal relationships in the structural model were mostly linear.

## 4. Results and Discussion

### 4.1. Demographic Characteristics of the Respondents

In this cross-sectional study, 154 males and 186 female students of the University of Malaya contributed and the average age was reported to be 24.32 years old (SD = 3.6 years). The students were similar with regard to their educational level and income (Table 2).

Table 3 shows an exploratory data analysis of the data set.

### 4.2. Convergent and Discriminant Validity

The results demonstrated that convergent validity exists for all constructs of the EBP, BSC, BA, and EES. Composite reliability (CR) larger than 0.7 is acceptable, and the average variance extracted (AVE) was ≥ 0.5 [49]. Most of the outer loadings were above 0.7 except one category of EBP, which was removed from the model. Moreover, the results of the heterotrait-monotrait ratio of the correlations methods showed that all the constructs had sufficient discriminant validity [50]. Finally, the number three exogenous variables (BA, BSC, and EES) and nine categories of EBP as endogenous constructs with their indicators remain in the model. After validation of the measurement model, the factor score of each latent variable was computed.

### 4.3. Path Analysis

**Figure 2:**
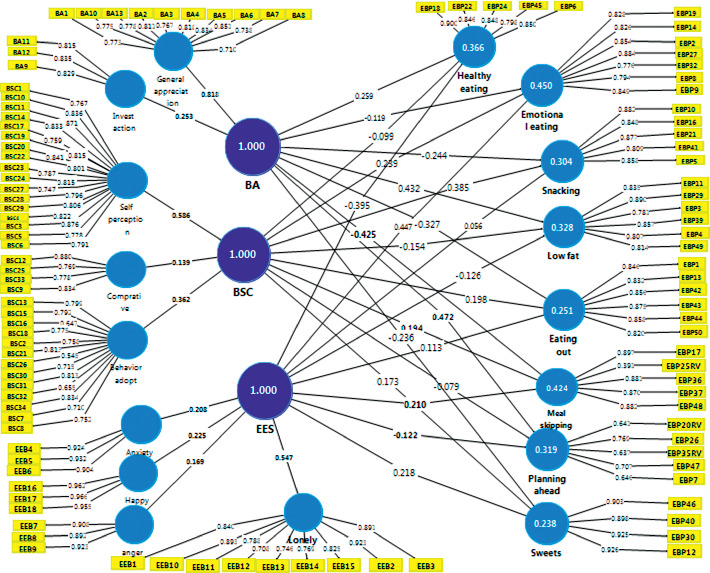
Path model.

Path analysis was used to check the causal relationship in the model. No multicollinearity was found among the constructs, and all outer weights were significant based on the results of bootstrapping. Seventeen constructs were known as reflective at the first order including three components of body shape concern, two components of body appreciation, four components of emotional eating scale, and eight categories of eating behavior patterns. All reflective constructs are shown in light blue in Figure 2. Also, BSC, BA, and EES were known as reflective constructs in the second order. Formative constructs are shown in dark blue in Figure 2. In this case, four constructs consisting of lonely/depression eating, anxiety eating, angry eating, and happy eating formed the emotional eating scale (EES). General appreciation and investment action behavior formed the body appreciation (BA), and finally body shape concern (BSC) was formed by self-perception, adaption behavior, and comparative behavior.

Figure 2 represents the path model. The number of 5000 samples was generated through bootstrapping. The result of the path analysis and PLS-SEM analysis (bootstrapping) indicated that only five of the path coefficients were not significant. The results of the bootstrapping method provided a *P* value for each path (Table 4). The structural model relationships were significant if the reported *P* value was less than 0.05. The outer loadings of each construct which is extracted from Figure 2 is shown in a separate figure.

### 4.4. The Coefficient of Determination *R*^2^

The value of *R*^2^ shows the contribution of BSC, EES, and BA to each endogenous construct. As can be seen, the exogenous constructs had the greatest contribution to emotional eating perception with adjusted *R*^2^ = 0.445. This value is followed by meal skipping at *R*^2^ = 0.418 and healthy eating with *R*^2^ = 0.360. The lowest contribution of exogenous variables was reported on sweets with the *R*^2^ = 0.231. The MSE for the model was calculated as well.

### 4.5. Hybrid Model (SEM-ANN)

At the last step, in order to conduct the hybrid model, the factor score calculated by SEM analysis for the body appreciation, body shape concern, and emotional eating scale was used as an input in the ANN model. Hence, the factor score of eight categories of eating behavior patterns (including low-fat eating, healthy eating, eating outsides, meal skipping, snaking, planning ahead, emotional eating, and sweets) was used as an output to the neural network model. The best number of nodes in hidden layer was chosen according to the criteria.

### 4.6. Criteria for Selection of ANN Architecture

This research used the ANN method [47], whereby the initial network was tested by considering 10, 11, 12, 13, 14, 15, 16, 17, 18, 19, and 20 hidden nodes. It was found that an optimal network with 17 hidden nodes has enough complexity to map the datasets without increasing the model's errors.

The input layer, therefore, consisted of three exogenous significant variables from the SEM (e.g., body appreciation, body shape concern, and emotional eating behavior), while the output layer consisted of eight dependent variables (e.g., eating outside, emotional eating, planning for food, meal skipping, healthy eating, low-fat eating, sweets, and snacking; cultural habit was eliminated due to low factor loading).

**Figure 3:**
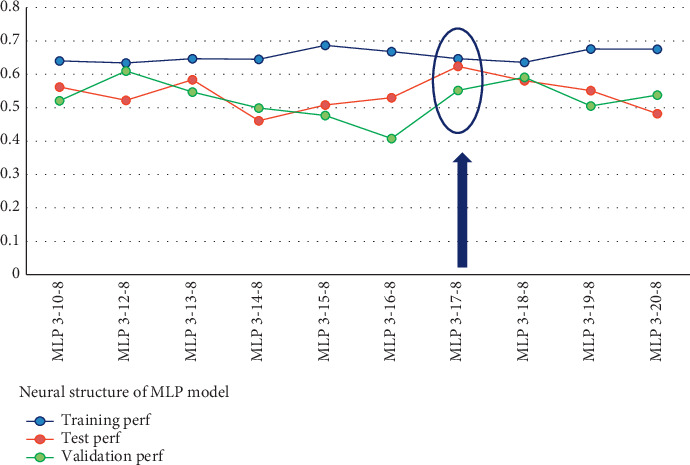
Network performance (*R*^2^).

Ten architectures were trained once more via backpropagation training algorithms to find the best algorithm for ANN training.  Figure 3 shows a neural network yielded from the analysis of Table 5. This is a feed-forward backpropagation network with neuron configuration of 3-17-8 with the training algorithm Levenberg–Marquardt (*trainlm*), with tan/sig as the transfer function for hidden layers and linear function for output. The Levenberg–Marquardt was proved to fit the best fit for the application (Table 5).

As can be seen in Figure 3, if the difference between training and test accuracy was the lowest, then our network is fit well at MLP 3-17-8. This result showed that the network model was quite reliable in capturing the numerical relations between the predictors and outputs.

From Table 6, the MSE for the training set was 0.548 while for the test set was 0.559. Therefore, the network model is quite reliable in capturing the numerical relations between the predictors and outputs.

**Figure 4:**
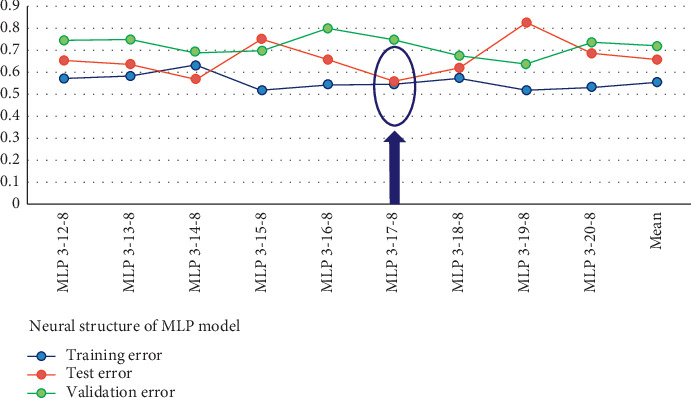
Network error MSE.

Considering the results of Table 6 and Figure 4, the training error is a bit lower than the corresponding test error. Therefore, it could be concluded that the optimal network happened at MLP 3-17-8.

**Figure 5:**
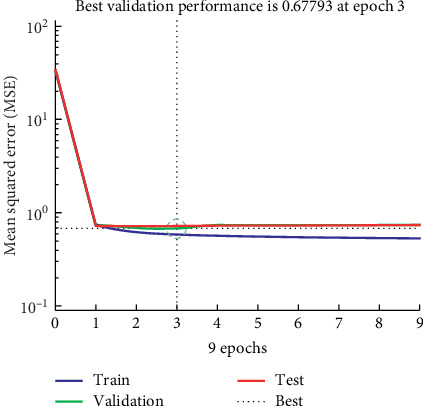
Validation performance for MLP 3-17-8.

The best validation performance was at epoch 3 with MSE = 0.677. Training was stopped at epoch 9.  Figure 5 shows the graphical validation network for MLP 3-17-8. The optimal number of iterations based on the performance of the validation set was found, which is clearly shown by the graph produced by MATLAB. In this work, the training data showed a good fit. *R* values were close to 0.6, so it explains more or less about 60% of the cases. It could be mentioned that hybrid methods have a good performance than typical statistics. However, further research is needed.

**Figure 6:**
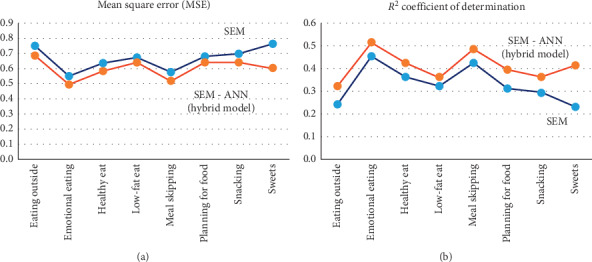
Comparison of (a) MSE and (b) *R*^2^ of both models.

In order to compare the prediction power of two techniques in terms of accuracy, *R*^2^ values and MSE of SEM and the hybrid model were compared. The result of Figure 6 showed that the value of *R*^2^ has increased through the hybrid model. In general, the hybrid model was shown to explain higher contribution of each independent variable to the dependent variable.

## 5. Discussion

This study is among the very few works that integrate the neural network and SEM [38, 51]. At first, it used SEM to check the internal consistency, convergent, and discriminant validity of variables through the SEM model. Then, the significant predictors in the model of eating behaviors were identified and incorporated into the ANN model. Similar to other studies [30, 38–41], this study has provided a way to develop the neural network model with a higher prediction performance by employing the results from the SEM. The results of these studies revealed that the hybrid model increased the prediction power of the model. However, the SEM technique exhibited good statistical properties, and the hybrid model improved the value of *R*^2^ (20%) while the amount of error was decreased (about 9.6%).

The findings indicated that SEM technique is quite robust in measuring the construct validity of the model. It was shown that all the constructs in the path model which were based on the questionnaire which had sufficient convergent and discriminant validity. Similar to other studies [52], the results indicated that the EBPQ has an adequate convergent and discriminant validity. Similar studies revealed adequate convergent and discriminant validity of the EESQ, but the factor structure of EESQ has not been assessed [17]. In addition, BASQ's construct validity is reported in the literature. These results support the findings of another study, which found a good distinction between these subscales of BAQ [53].

According to the results of SEM analysis, most of the relationships were significant between body shape concern, body appreciation, and emotional eating scale (as three independent variables) and eight categories of eating behavior patterns (eating outside, emotional eating, meal skipping, snaking, sweets, low-fat eating, healthy eating, and planning for food).

The findings revealed that the effect of body shape concern on the subscales of eating behavior patterns is significant, as an increase in BSC directly causes a proportionate increase in unhealthy eating patterns especially in eating outside, emotional eating, meal skipping, snacking, and sweets. Similar results were obtained in a study of body shape concerns and eating behaviors among Indian urban adolescent girls [54]. Extensive research indicates that body shape concern predicts unhealthy eating behaviors and leading to eating disorders [55, 56]. The body shape concern does not have a significant association with healthy eating and planning food.

Furthermore, an increasing body appreciation results in an increase in healthy eating, low-fat eating, and planning for food, whereas the same increase in body appreciation resulted in a decrease in eating outside, emotional eating, meal skipping, snacking, and sweets. However, no significant direct relationship was found between body appreciation and emotional eating in another study [57].

Generally, emotional eating is defined as eating in reaction to negative emotions which is related to the avoidance of unpleasant sensations and feelings. Our result was in line with other results which indicated that both positive and negative emotion play important roles in eating behaviors [58]. It can be seen that there is an increase in emotional eating, the emotional eating perception, sweets, and meal skipping. Previous studies showed that the participants were more likely to consume snacks and have high-energy intake of carbohydrate and fat where they were emotionally invalided in eating [59, 60]. Moreover, the emotional eating scale was significantly associated with emotional eating, sweets, meal skipping, healthy eating, and low-fat eating.

The SEM technique showed good statistical properties. However, the results illustrated that the hybrid model is superior to SEM in prediction [61]. The hybrid model used in this study is not only applicable to predict the eating behavior, but it is also quite a robust method that could be applied in modeling the public health and social science problems. This research provides a new viewpoint in understanding hybrid models, which is an important extra contribution to the available literature because artificial neural networks are used. In fact, a multianalytic method is introduced by integrating neural networks and PLS-SEM.

## 6. Conclusion

The present study is an empirical examination of the eating behavior pattern, while the model supported the traditional theories together with the new methods. In fact, it was shown that using the hybrid model (SEM-ANN) was suitable to assess the relationship between the EES, BSC, BA, and EBP due to the ambiguity derived from having both linear and nonlinear aspects. ANN and SEM approaches complement each other. This is quite a new method that rarely has been used in the field of public health. The method enables researchers to evaluate the model with a higher level of *R*^2^ and lower MSE.

### 6.1. Limitation and Recommendation

ANN analysis has its own limitations such as the black-box operating nature [62]. Long training times is another limitation of the ANN techniques. Sometimes at least 100 iterations are required to train the simple network. Moreover, research should be done with a more complex model and even apply more traditional statistics to hybrid with neural networks. In addition, other neural networks such as a genetic algorithm or machine learning methods should be applied.

As far as the participants of the study (i.e., university students) are concerned, they may not represent clinical patients or adult population. Also, future studies could focus on effect of age level on eating behavior and more homogenous sample could be considered. Moreover, considering the fact that the concept of body appreciation does not share the same conceptual and factorial structure across different cultures, it is recommended that the validity and the reliability of this instrument across different cultures be evaluated.

## Figures and Tables

**Table 1:** Literature review of SEM-ANN research.

No	Research	Year	Hybrid model	Case study input	Evaluation criteria	Results and discussion	Statistical model
1	[37]	2016	SEM-ANN	Students' intention towards academic use of Facebook	*R* ^2^ RMSEA	The hybrid model helps to better understand factors that predict the usage of Facebook in higher education	CB-SEM
2	[30]	2015	SEM-ANN	Influence of SERVPERF on customer satisfaction and customer loyalty among low cost and full service	RMSEA	The use of the two-stage predictive-analytic SEM-neural network analysis may provide a more holistic understanding and thus may provide a significant methodological contribution from the statistical point of view	CB-SEM
3	[38]	2013	SEM-ANN	Factors that influence consumers' mobile-commerce adoption intention	RMSEA	Employing a multianalytic approach demonstrated how combining two different data analysis approaches in either methodology and the alternative analysis is able to improve the validity and confidence in the results	CB-SEM
4	[39]	2012	SEM-ANN	Adoption of an interorganizational system standard and its benefits by using RosettaNet	RMSEA	Improved existing technology adoption methodology was achieved by integrating both SEM and neural network for examining the adoptions of RosettaNet	CB-SEM
5	[40]	2014	SEM-ANN	User's intention to adopt mobile learning, Malaysia	RMSEA	This has provided a novel perspective in examining the key determinants of m-learning acceptance, while a greater amount of variance was explained in this model	CB-SEM
6	[41]	2014	SEM-ANN	Predictors of open interorganizational systems (IOS) adoption by using RosettaNet as a case study	RMSE	The neural network supports the antecedents of RosettaNet adoption in SMEs	PLS-SEM
7	[42]	2019	SEM-ANN	Predict customers' intention to purchase battery electric	RMSE	A new approach solved the analytical problems in this research field	PLS-SEM

**Table 2:** Demographic characteristics.

	Mean	SD
Age	24.32	3.6
	Range (22–36 years)	
BMI	23.8	4.95
	Range (15.98–41.69)	
	Frequency	%
Gender		
Male	154	45.29
Female	186	54.71
Education level		
Diploma	2	0.59
Baccalaureate	234	68.82
Doctor of philosophy	89	26.18
Postdoctoral	15	4.41
Total	340	100

**Table 3:** Exploratory data analysis of the dataset.

Item	Mean	SD
*EBP*		
Healthy eating	3.093	0.859
Emotional eating	2.832	0.934
Snacking	2.799	0.87
Eating outside	2.794	0.908
Planning for food	2.980	0.519
Low-fat eating	2.953	0.702
Cultural habit	3.015	0.956
Sweets	2.818	0.861
Meal skipping	2.82	0.734
*BSC*		
Self-perception	3.386	1.237
Behavior adapt	2.545	1.091
Comparative behavior	3.315	1.207
*BA*		
General appreciation	3.885	0.978
Investment action	3.643	1.044
*EES*		
Depression/bored/lonely eating	2.649	1.042
Anxiety eating	2.658	1.099
Angry eating	2.51	0.98
Happy eating	2.863	1.252

**Table 4:** Results of bootstrapping for path analysis.

Path	BSC		BA		EES	
EBP categories	Beta	*P* values	Beta	*P* values	Beta	*P* values
Eating out	0.198	≤0.01	−0.327	≤0.01	0.113	0.07
Emotional eating	0.239	≤0.01	−0.119	≤0.05	0.447	≤0.01
Healthy eating	−0.099	0.101	0.259	≤0.01	−0.395	≤0.01
Low fat	−0.154	≤0.01	0.432	≤0.01	−0.126	≤0.05
Meal skipping	0.194	≤0.01	−0.425	≤0.01	0.211	≤0.01
Planning ahead	−0.079	0.212	0.472	≤0.01	−0.122	0.08
Snacking	0.385	≤0.01	−0.244	≤0.01	0.056	0.34
Sweets	0.173	≤0.01	−0.236	≤0.01	0.218	≤0.01

BSC: body shape concern, BA: body appreciation, and EES: emotional eating scale.

**Table 5:** Network performance (*R*^2^).

No	Design MLP input-hidden-output	Training perf	Test perf	Validation perf
1	MLP 3-10-8	0.640	0.562	0.521
2	MLP 3-12-8	0.634	0.522	0.610
3	MLP 3-13-8	0.647	0.584	0.547
4	MLP 3-14-8	0.645	0.461	0.499
5	MLP 3-15-8	0.687	0.508	0.477
6	MLP 3-16-8	0.668	0.530	0.408
7	**MLP 3-17-8**	**0.647**	**0.624**	**0.552**
8	MLP 3-18-8	0.636	0.581	0.591
9	MLP 3-19-8	0.676	0.551	0.505
10	MLP 3-20-8	0.675	0.482	0.538

**Table 6:** Network error (MSE).

No	Design MLP input-hidden-output	Training error	Test error	Validation error
1	MLP 3-10-8	0.550	0.730	0.726
2	MLP 3-12-8	0.571	0.653	0.747
3	MLP 3-13-8	0.584	0.636	0.750
4	MLP 3-14-8	0.634	0.571	0.692
5	MLP 3-15-8	0.520	0.750	0.701
6	MLP 3-16-8	0.547	0.661	0.799
7	**MLP 3-17-8**	**0.548**	**0.559**	**0.751**
8	MLP 3-18-8	0.577	0.623	0.677
9	MLP 3-19-8	0.518	0.830	0.640
10	MLP 3-20-8	0.534	0.685	0.738

## Data Availability

The Numeric data-based questionnaire used to support the findings of this study are included within the supplementary information file. Also, the Excel sheet of raw data was used for analysis and supported the findings of this study.

## Supplementary Materials

Supplementary Materials Excel data sheet.

## References

[1] Chong M. F.-F., Ayob M. N. M., Chong K. J. (2016). Psychometric analysis of an eating behaviour questionnaire for an overweight and obese Chinese population in Singapore. *Appetite*.

[2] Partridge S. R., McGeechan K., Bauman A., Phongsavan P., Allman-Farinelli M. (2016). Improved eating behaviours mediate weight gain prevention of young adults: moderation and mediation results of a randomised controlled trial of TXT2BFiT, mHealth program. *International Journal of Behavioral Nutrition and Physical Activity*.

[3] Sutin A., Robinson E., Daly M., Terracciano A. (2016). Weight discrimination and unhealthy eating-related behaviors. *Appetite*.

[4] Schifter D. E., Ajzen I. (1985). Intention, perceived control, and weight loss: an application of the theory of planned behavior. *Journal of Personality and Social Psychology*.

[5] Schlundt D. G., Hargreaves M. K., Buchowski M. S. (2003). The eating behavior patterns questionnaire predicts dietary fat intake in African American women. *Journal of the American Dietetic Association*.

[6] Stojek M. M. K., Tanofsky-Kraff M., Shomaker L. B. (2017). Associations of adolescent emotional and loss of control eating with 1-year changes in disordered eating, weight, and adiposity. *International Journal of Eating Disorders*.

[7] Sultson H., Kukk K., Akkermann K. (2017). Positive and negative emotional eating have different associations with overeating and binge eating: construction and validation of the Positive-Negative Emotional Eating Scale. *Appetite*.

[8] Van Strien T., Konttinen H., Homberg J. R., Engels R. C. M. E., Winkens L. H. H. (2016). Emotional eating as a mediator between depression and weight gain. *Appetite*.

[9] Beaudin L., Skaza J. (2015). Measuring the total impact of demographic and behavioural factors on the risk of obesity accounting for the depression status: a structural model approach using new BMI. *Applied Economics*.

[10] King-Kallimanis B. L., Oort F. J., Visser M. R. M., Sprangers M. A. G. (2009). Structural equation modeling of health-related quality-of-life data illustrates the measurement and conceptual perspectives on response shift. *Journal of Clinical Epidemiology*.

[11] Gailledrat L., Rousselet M., Venisse J.-L. (2016). Marked body shape concerns in female patients suffering from eating disorders: relevance of a clinical sub-group. *PLoS One*.

[12] da Silva W. R., Dias J. C. R., Maroco J., Campos J. A. D. B. (2014). Confirmatory factor analysis of different versions of the Body Shape Questionnaire applied to Brazilian university students. *Body Image*.

[13] Andrew R., Tiggemann M., Clark L. (2016). Predicting body appreciation in young women: an integrated model of positive body image. *Body Image*.

[14] Avalos L., Tylka T. L., Wood-Barcalow N. (2005). The body appreciation scale: development and psychometric evaluation. *Body Image*.

[15] Holmqvist K., Frisén A. (2012). “I bet they aren’t that perfect in reality”: Appearance ideals viewed from the perspective of adolescents with a positive body image. *Body Image*.

[16] Bongers P., de Graaff A., Jansen A. (2016). “Emotional” does not even start to cover it: generalization of overeating in emotional eaters. *Appetite*.

[17] Schneider K. L., Panza E., Appelhans B. M., Whited M. C., Oleski J. L., Pagoto S. L. (2012). The emotional eating scale. Can a self-report measure predict observed emotional eating?. *Appetite*.

[18] Johnson F., Pratt M., Wardle J. (2012). Dietary restraint and self-regulation in eating behavior. *International Journal of Obesity*.

[19] Hsu S.-H., Chen W.-H., Hsieh M.-J. (2006). Robustness testing of PLS, LISREL, EQS and ANN-based SEM for measuring customer satisfaction. *Total Quality Management & Business Excellence*.

[20] Hair J. F., Ringle C. M., Sarstedt M. (2011). PLS-SEM: indeed a silver bullet. *Journal of Marketing Theory and Practice*.

[21] Demir A., Shadmanov A., Aydinli C., Okan E. (2015). Designing a forecast model for economic growth of Japan using competitive (hybrid ANN vs multiple regression) models. *Ecoforum Journal*.

[22] Guarnaccia C., Quartieri J., Tepedino C. A hybrid predictive model for acoustic noise in urban areas based on time series analysis and artificial neural network.

[23] Panigrahi S., Behera H. S. (2017). A hybrid ETS-ANN model for time series forecasting. *Engineering Applications of Artificial Intelligence*.

[24] Zhu Y., Xie C., Sun B., Wang G.-J., Yan X.-G. (2016). Predicting China’s SME credit risk in supply chain financing by logistic regression, artificial neural network and hybrid models. *Sustainability*.

[25] Saba T., Rehman A., AlGhamdi J. S. (2017). Weather forecasting based on hybrid neural model. *Applied Water Science*.

[26] Mohamad E. T., Faradonbeh R. S., Armaghani D. J., Monjezi M., Majid M. Z. A. (2017). An optimized ANN model based on genetic algorithm for predicting ripping production. *Neural Computing and Applications*.

[27] Yu P., Low M. Y., Zhou W. (2018). Development of a partial least squares-artificial neural network (PLS-ANN) hybrid model for the prediction of consumer liking scores of ready-to-drink green tea beverages. *Food Research International*.

[28] Yazdani-Chamzini A., Zavadskas E., Antucheviciene J., Bausys R. (2017). A model for shovel capital cost estimation, using a hybrid model of multivariate regression and neural networks. *Symmetry*.

[29] Shafaei M., Adamowski J., Fakheri-Fard A., Dinpashoh Y., Adamowski K. (2016). A wavelet-SARIMA-ANN hybrid model for precipitation forecasting. *Journal of Water and Land Development*.

[30] Leong L.-Y., Hew T.-S., Lee V.-H., Ooi K.-B. (2015). An SEM-artificial-neural-network analysis of the relationships between SERVPERF, customer satisfaction and loyalty among low-cost and full-service airline. *Expert Systems with Applications*.

[31] Rigdon E. E., Ringle C. M., Sarstedt M., Naresh K. M. (2010). Structural modeling of heterogeneous data with partial least squares. *Review of Marketing Research*.

[32] Wang L., Zou H., Su J., Li L., Chaudhry S. (2013). An ARIMA-ANN hybrid model for time series forecasting. *Systems Research and Behavioral Science*.

[33] Papaioannou G., Dikaiakos C., Dramountanis A., Papaioannou P. (2016). Analysis and modeling for short- to medium-term load forecasting using a hybrid manifold learning principal component model and comparison with classical statistical models (SARIMAX, exponential smoothing) and artificial intelligence models (ANN, SVM): the case of Greek electricity market. *Energies*.

[34] Iliadis L., Anezakis V.-D., Demertzis K., Mallinis G. (2017). Hybrid unsupervised modeling of air pollution impact to cardiovascular and respiratory diseases. *International Journal of Information Systems for Crisis Response and Management*.

[35] Ghritlahre H. K. (2019). Performance prediction of porous bed solar air heater using MLP and GRNN model-a comparative study. *CSVTU Research Journal on Engineering and Technology*.

[36] Rath J. S., Hutton P. H., Chen L., Roy S. B. (2017). A hybrid empirical-Bayesian artificial neural network model of salinity in the San Francisco Bay-Delta estuary. *Environmental Modelling and Software*.

[37] Sharma S. K., Joshi A., Sharma H. (2016). A multi-analytical approach to predict the Facebook usage in higher education. *Computers in Human Behavior*.

[38] Chong A. Y.-L. (2013). A two-staged SEM-neural network approach for understanding and predicting the determinants of m-commerce adoption. *Expert Systems with Applications*.

[39] Chan F. T. S., Chong A. Y. L. (2012). A SEM-neural network approach for understanding determinants of interorganizational system standard adoption and performances. *Decision Support Systems*.

[40] Tan G. W.-H., Ooi K.-B., Leong L.-Y., Lin B. (2014). Predicting the drivers of behavioral intention to use mobile learning: a hybrid SEM-neural networks approach. *Computers in Human Behavior*.

[41] Chong A. Y.-L., Bai R. (2014). Predicting open IOS adoption in SMEs: an integrated SEM-neural network approach. *Expert Systems with Applications*.

[42] Xu Y., Zhang W., Bao H., Zhang S., Xiang Y. (2019). A SEM-neural network approach to predict customers’ intention to purchase battery electric vehicles in China’s Zhejiang province. *Sustainability*.

[43] Cohen L., Manion L., Morrison K. (2007). *Research Methods in Education*.

[44] Soper D. S. (2018). A-priori sample size calculator for structural equation models. http://www.danielsoper.com/statcalc.

[45] Cooper P. J., Taylor M. J., Cooper Z., Fairbum C. G. (1987). The development and validation of the body shape questionnaire. *International Journal of Eating Disorders*.

[46] Arnow B., Kenardy J., Agras W. S. (1995). The Emotional Eating Scale: the development of a measure to assess coping with negative affect by eating. *International Journal of Eating Disorders*.

[47] Sexton R. S., Johnson R. A., Hignite M. A. (2002). Predicting Internet/e-commerce use. *Internet Research*.

[48] Piotrowski A. P., Napiorkowski J. J. (2011). Optimizing neural networks for river flow forecasting—Evolutionary Computation methods versus the Levenberg-Marquardt approach. *Journal of Hydrology*.

[49] Hair J. F., Hult G. T. M., Ringle C., Sarstedt M. (2016). *A Primer on Partial Least Squares Structural Equation Modeling (PLS-SEM)*.

[50] Henseler J., Ringle C. M., Sarstedt M. (2015). A new criterion for assessing discriminant validity in variance-based structural equation modeling. *Journal of the Academy of Marketing Science*.

[51] Hsu C.-I., Shih M.-L., Huang B.-W., Lin B.-Y., Lin C.-N. (2009). Predicting tourism loyalty using an integrated Bayesian network mechanism. *Expert Systems with Applications*.

[52] Dehghan P., Asghari-Jafarabadi M., Salekzamani S. (2015). Validity, reliability and feasibility of the eating behavior pattern questionnaire (EBPQ) among Iranian female students. *Health promotion perspectives*.

[53] Swami V., Chamorro-Premuzic T. (2008). Factor structure of the body appreciation scale among Malaysian women. *Body Image*.

[54] Som N., Mukhopadhyay S. (2015). Body weight and body shape concerns and related behaviours among Indian urban adolescent girls. *Public Health Nutrition*.

[55] Martins C. R., Carraça E., Teixeira P. J., Silva A. M., Petroski E. L. P. (2014). Prevalence of body shape concerns and associated factors among Brazilian early adolescents. *Human Movement*.

[56] Perini T. A., Vieira R. S., Vigário P. D. S., Oliveira G. L. D., Ornellas J. D. S., Oliveira F. P. D. (2009). Transtorno do comportamento alimentar em atletas de elite de nado sincronizado. *Revista Brasileira de Medicina do Esporte*.

[57] Oswald A., Chapman J., Wilson C. (2017). Do interoceptive awareness and interoceptive responsiveness mediate the relationship between body appreciation and intuitive eating in young women?. *Appetite*.

[58] Zhu H., Cai T., Chen G., Zhang B. (2013). Validation of the emotional eating scale among Chinese undergraduates. *Social Behavior and Personality: An International Journal*.

[59] Costarelli V., Patsai A. (2012). Academic examination stress increases disordered eating symptomatology in female university students. *Eating and Weight Disorders-Studies on Anorexia, Bulimia and Obesity*.

[60] Lemmens S. G., Rutters F., Born J. M., Westerterp-Plantenga M. S. (2011). Stress augments food “wanting” and energy intake in visceral overweight subjects in the absence of hunger. *Physiology & Behavior*.

[61] Scott J. E., Walczak S. (2009). Cognitive engagement with a multimedia ERP training tool: assessing computer self-efficacy and technology acceptance. *Information & Management*.

[62] Sim J.-J., Tan G. W.-H., Wong J. C. J., Ooi K.-B., Hew T.-S. (2014). Understanding and predicting the motivators of mobile music acceptance-a multi-stage MRA-artificial neural network approach. *Telematics and Informatics*.

